# Subjective health complaints in Norwegian adolescents and associations with stress, sex, and age – Cross-sectional trends over a 10-year period

**DOI:** 10.1177/14034948251357426

**Published:** 2025-11-06

**Authors:** Unni K. Moksnes, Magdalena Lazarewicz, Geir Arild Espnes

**Affiliations:** 1 Department of Public Health and Nursing, Norwegian University of Science and Technology / NTNU Center for Health Promotion Research, Trondheim, Norway; 2 Department of Health Psychology, Medical University of Warsaw, Warsaw, Poland

**Keywords:** Psychosomatic symptoms, youth, stressors

## Abstract

*Aims:* This study investigated cross-sectional trends in subjective health complaints (SHCs) over a 10-year period and explored associations between sex, age, normative stress domains, and SHCs. *Methods:* Data were drawn from three cross-sectional surveys in 2011 (*n* = 1,289), 2016 (*n* = 1,233), and 2022 (*n* =310), including adolescents aged 13–20 from lower- and upper-secondary public schools in rural municipalities in Mid-Norway. SHCs were assessed using a 12-item scale measuring physical and mental symptoms, while stress was measured with the 30-item Adolescent Stress Questionnaire. Descriptive and multiple linear regression analyses were conducted. *Results:* Average SHC scores were moderately high, showing a slight increase over the study period, and were consistently higher in girls than boys. Age showed a curvilinear association with SHCs, with increasing SHC levels from ages 13 to 16 and declining levels from ages 17 to 20. All stressors associated significantly positively with SHCs controlled for sex and age. Stressors related to the school context were significantly positively associated with SHCs controlled for all covariates, alongside a significant interaction effect of sex by school attendance. ***Conclusions:*** **The study shows a relatively stable level of SHCs in adolescents, with a minor increase in average levels over the 10 years investigated. Girls reported higher SHCs than boys and a curvilinear association was found between age and SHCs. The findings emphasize the significant influence of especially school-related stressors on adolescent SHCs.**

## Background

Ensuring equitable opportunities for youth to achieve positive development, health, and well-being is a shared public health goal [1]. Therefore, understanding adolescents’ self-perceived health and identifying key factors impacting on health and well-being are essential for fostering positive development in adolescence [1]. While most adolescents in high-income countries like Norway with relatively small socio-economic differences enjoy good health [1, 2], symptoms such as unexplained musculoskeletal pain and headaches significantly impact their functioning and well-being [3, 4]. Subjective health complaints (SHCs), or ‘psychosomatic symptoms’, which encompass a range of mental (e.g. nervousness, sleeping difficulties, depressive symptoms) and physical (e.g. headache, backache) symptoms lacking clear medical explanation [5, 6], are widespread and often co-occur [7, 8]. A range of studies show that girls report higher symptom levels than boys [4, 8–11] and that symptoms seem to increase with age during adolescence, especially for females [11, 12, 13]; however, findings are not consistent.

A Norwegian study reported an increasing trend in the level of SHCs among adolescents between 1994 and 2014, particularly among older adolescent girls [11]. However, recent national reports from Ungdata (Young Data) covering the period 2021–2024 indicate relatively stable levels of SHCs, with 12% of adolescents reporting daily physical symptoms such as nausea and pain, and 46% reporting experiencing these symptoms ‘sometimes’ [2]. Internationally, trends in levels of SHCs vary across countries [14], but an increasing trend in SHCs has been observed in Nordic and northern European nations during the last decades [4, 7, 11, 13, 15]. The last decade has furthermore shown a strong increase in the prevalence of symptoms of depression and anxiety among young people, especially among girls [16, 17]. The rise in SHCs has been linked to stress, a common experience in adolescence [18, 19].

Stress is a key factor associated with SHCs and can be defined as a perceived mismatch between demands – whether real or imagined – and available resources to cope effectively [20]. Adolescents face numerous stressors, such as academic pressures, school–leisure conflicts, and challenges in interpersonal relationships with peers, parents, teachers, and romantic partners [21, 22]. Perceived stress thus tends to increase in intensity and frequency during adolescence [23], with girls experiencing higher stress levels and greater susceptibility to its negative health effects than boys [24, 25]. Notably, stress accumulation is linked to SHCs [18, 19]; yet research on how different normative stressors associate with SHCs during adolescence remains limited. The COVID-19 pandemic introduced additional stressors for adolescents, including social isolation, disruptions to daily routines, and uncertainty about the future, all of which have been linked to increased psychological distress and SHCs [26]. While the pandemic itself is considered a non-normative stressor due to its unexpected nature, the widespread challenges it introduced temporarily became part of adolescents’ shared lived experiences [26].

Adolescence is a critical developmental stage characterized by rapid psychosocial, emotional, and physiological changes that form the foundation for long-term health trajectories [27]. This period is particularly important for investigating SHCs, as research has consistently shown that adolescence is associated with the emergence and recurrence of both somatic and psychological symptoms and functional impairment [4, 7, 11, 28], highlighting the importance of early health surveillance and intervention during adolescence. A systematic review and meta-analysis have demonstrated that temporal trends in psychosomatic complaints among adolescents reflect broader public health concerns, underscoring the need for continuous monitoring to identify emerging determinants of SHCs [7]. Additionally, adolescence is a key period for the development of health-related behaviours that frequently persist into adulthood [27]. Given that adolescence is shaped by substantial personal and contextual changes and plays a critical role in establishing positive health development from a life course perspective, investigating the influence of normative stressors on SHCs is essential to understanding how everyday stressors affect adolescents’ subjective health [7, 18, 19]. Therefore, the present study set out to examine:

(a) sex and age differences in SHCs,
(b) cross-sectional trends in SHCs over a 10-year period – including the years of the COVID-19 pandemic – and
(c) associations between different stress domains and SHCs.

## Material and methods

### Procedures

This study is based on data from the project ‘Living in rural communities’, conducted every five years since 1996 in public lower- and upper-secondary schools located in inland and coastal rural municipalities in the southern part of Trøndelag County, Mid-Norway. The project focuses on adolescents’ experiences of daily life contexts, including school, home environment, the local community, leisure activities, health, and well-being. Accordingly, this study uses a repeated cross-sectional design, with data collected in 2011, 2016, and 2022 from independent samples of adolescents in the same rural communities, using identical questionnaires. Data from 2011 and 2016 included five municipalities, while 2022 data included four municipalities (selected based on their participation in previous data collections).

Prior to data collection, schools were contacted regarding participation in the study. Once the municipality authorities and school principals approved participation, an information letter was sent to students and to the parents of those under 16 years of age. Students aged 16 years and older provided consent by completing the questionnaire, while written parental consent was required for students aged 13–15 years. Paper questionnaires were used in 2011 and 2016 data collection, while the 2022 data were collected using an online format. All data collections were administrated in classrooms during 45-minute sessions, with teacher assistance. Non-participating students were allowed to engage in other schoolwork.

The study was approved by the Regional Committee for Medical Research Ethics Mid-Norway (approval number 2016/1165).

### Participants

Participants were included based on being students in schools taking part in the study. All students present on the day of data collection, who had provided parental or own consent (depending on age), were invited to participate.

### 2011 sample

A total of 1924 students were invited to participate, with 1289 completing the questionnaire (67% response rate). In the sample, 654 were girls and 630 were boys, 5 undisclosed). Nonresponses were mainly due to student absences or refusals to participate. Participants’ ages ranged from 13 to 20 years.

### 2016 sample

Of the 1,906 students invited, 1,282 completed the questionnaire (67% response rate). Restricting the sample to ages 13–20 reduced the sample size to 1,233 (580 girls and 644 boys, 9 undisclosed).

### 2022 sample

A total of 1,538 students were invited to participate, with 310 completing the questionnaire (20.2 response rate). The final sample of 310 included 166 (53.5) girls and 141 (45.3) boys, with 3 undisclosed. Ages ranged from 13 to 20 years.

### Measures

SHCs were measured using 12 items assessing self-reported physical symptoms (e.g. stomachache, headache, back or limb pain, cold, asthma) and mental symptoms (e.g. bad mood, loneliness, nervousness, sadness, or irritability) experienced during the past four weeks [29]. Responses were rated by the participating adolescents on a four-point scale ranging from 1 ‘not bothered’ to 4 ‘very much bothered’. Items were computed as a sum score ranging from 12 to 48, with higher scores indicating greater symptom load.

*Stress* was assessed with the Norwegian version of the Adolescent Stress Questionnaire (ASQ-N) [22], measuring normative adolescent stressors. The scale includes 30 items divided into seven dimensions: (1) school performance (e.g. ‘*Having to study things you do not understand*’), (2) school/leisure conflict (e.g. ‘*Not enough time for leisure activities*’), (3) peer pressure (e.g. ‘*Being hassled for not fitting in*’), (4) home life (e.g. ‘*Disagreements between you and your mother or father*’), (5) romantic relationships (e.g. ‘*Not enough time for your boyfriend/girlfriend*’), (6) teacher/adult interactions (e.g. ‘*Not being listened to by teachers*’), and (7) school attendance (e.g. ‘*Compulsory school attendance*’). Responses are rated on a five-point scale ranging from 1 ‘not at all stressful or irrelevant’ to 5 ‘very stressful’, with higher scores indicating higher stress levels. The ASQ-N has been validated in Norwegian adolescents, indicating adequate psychometric properties [22].

*Self-rated health* was measured with a single item: ‘*How is your health at the moment*?’ Response values ranged from 1 ‘very bad’ to 5 ‘very good’. This item has been validated as a general health indicator in adolescents [30].

*Self-esteem* was assessed with the Rosenberg Self-Esteem Scale (RSE) [31], a 10-item scale with responses ranging from 1 ‘strongly disagree’ to 4 ‘strongly agree’. A higher sum score indicates higher self-esteem. Examples of items included in the scale are: ‘*On the whole, I am satisfied with myself*’, ‘*I wish I could have more respect for myself*’, ‘*I take a positive attitude toward myself*’, and ‘*At times I think I am no good at all*’. The RSE has been previously validated among Norwegian adolescents as a measure of global self-esteem [31].

*Leisure time physical activity* was measured with one item: ‘*During the last four weeks, how many days a week have you engaged in sports or physical activity so hard that you had high respiratory frequency, sweated, or had an increased heart rate for 20 minutes (or more)?*’ Responses ranged from 1 ‘never’ to 5 ‘most days per week’.

### Statistical analyses

Statistical analyses were conducted using SPSS 27.0 and Stata 17. Descriptive statistics included presentation of distribution of sex and age groups across the three cross-sectional samples. Mean scores were calculated for the stress dimensions, self-rated health, self-esteem, and physical activity across the cross-sectional samples. Multiple linear regression was used to investigate associations between the main study variables: sex, age, time point, stressor domains, and SHCs. First, we analysed associations between sex, age, time point, and SHCs in a multiple linear regression model. Associations between age and SHCs were investigated with reference to curvilinear associations. Time was investigated as a dummy variable, with 2011 as the reference category. To investigate the association between stressor domains and SHCs, each individual stressor domain was first investigated in association with SHCs (Model 1), controlling for sex and age. All stressor domains were included in Model 2, controlling for sex, age, time, self-rated health, physical activity, and self-esteem. Interaction effects were tested with interaction terms including sex and each stress domain.

Model assumptions were tested, with variation inflation factor (VIF) values between 1.10 and 2.83 (average VIF = 1.94), indicating no multicollinearity issues [32]. The Breusch–Pagan test indicated heteroscedasticity; however, no serious violations were indicated due to the large sample size where the scatter plot displayed a more random pattern of residuals. Missing values for stress and SHC variables ranged from 3.5 to 11.9%. Cases with up to 20% missing responses were included in the scale sum scores. Listwise deletion for missing cases was applied in the multivariate linear regression analyses. In Model 1, investigating associations between sex, age, time, and SHCs, the sample size was *n* = 2,108. In the regression models testing each individual stressor in association with SHCs controlled for sex and age, sample sizes ranged between *n* = 1,980 and *n* = 2,064 (Model 1). In Model 2, including all stressors in association with SHCs, sample size was *n* = 1,810. *P*-values ⩽ 0.05 were considered statistically significant.

## Results

### Descriptive statistics

Table I presents demographic characteristics across measurement points, showing an even distribution of sexes but greater variation in age groups. Mean SHC scores (Table II) remained slightly above the mid-point of the scale at all time points, with the highest scores observed in 2022, followed by 2016, and the lowest in 2011. SHC scores were consistently higher among girls compared to boys. Across age groups, the highest mean SHC scores were found among 15–16-year-olds in 2011, and among 17–20-year-olds in 2016 and 2022. Mean scores on the different stress scales were also close to the mid-point of the scale across all three time points but were highest in 2011. The highest mean scores were observed for stress related to peer pressure, school performance, and school/leisure conflict.

**Table I.** Demographic characteristics for the adolescent sample aged 13–20 years.

	2011	2016	2022	Total
	*n* (%)	*n* (%)	*n* (%)	*n* (%)
Sex				
Girls	654 (50.7)	580 (47.0)	166 (53.5)	1400 (49.4)
Boys	630 (48.9)	644 (52.2)	141 (45.5)	1415 (50.0)
Missing	5 (0.4)	9 (0.7)	3 (1.0)	17 (0.6)
Age				
13–14	540 (41.9)	381 (30.9)	58 (18.7)	979 (34.6)
15–16	430 (33.4)	453 (36.7)	121 (39.0)	1004 (35.4)
17–20	269 (20.9)	399 (32.4)	125 (40.3)	793 (28.0)
Missing	50 (3.8)		6 (1.9)	56 (2.0)
Total	1289 (100)	1233 (100)	310 (100)	2832 (100)

**Table II.** Mean scores on subjective health complaints, stressor domains, self-rated health, self-esteem, and physical activity across time points.

	Mean (SD)				Min / max	Cronbach`s α
	2011 (*n* = 1,289)	2016 (*n* = 1,233)	2022 (*n* = 310)	Total		
Subjective health complaints	18.87 (5.44)	20.00 (5.52)	21.43 (6.70)	19.59 (5.71)	12–48	0.88
Sex						
Girls	20.45 (5.48)	21.64 (5.82)	23.48 (6.69)	21.26 (5.85)		
Boys	17.08 (4.81)	18.28 (4.62)	18.83 (5.65)	17.72 (4.90)		
Age						
13–14	18.25 (5.02)	19.08 (5.07)	19.87 (6.29)	18.64 (5.15)		
15–16	19.74 (5.91)	20.16 (5.54)	21.68 (6.49)	20.20 (5.90)		
17–20	18.94 (5.46)	20.96 (5.91)	22.49 (6.70)	20.33 (6.11)		
Stress domains						
Home life	9.98 (4.60)	8.80 (4.07)	7.77 (3.83)	9.31 (4.41)	5–25	0.83
Romantic relationships	7.75 (4.72)	6.55 (4.04)	5.55 (3.41)	7.08 (4.41)	4–20	0.88
School attendance	8.47 (3.57)	8.11 (3.41)	7.55 (3.48)	8.23 (3.52)	4–20	0.69
School/leisure conflict	10.38 (4.36)	9.08 (4.04)	8.03 (3.93)	9.63 (4.29)	4–20	0.81
School performance	10.07 (4.05)	9.70 (4.01)	8.56 (4.05)	9.77 (4.05)	4–20	0.83
Teacher/adult interaction	7.64 (4.12)	6.48 (3.57)	5.39 (2.52)	6.97 (3.86)	4–20	0.87
Peer pressure	11.03 (4.97)	9.59 (4.55)	8.78 (4.03)	10.26 (4.79)	5–25	0.82
Self-rated health	3.78 (0.97)	3.25 (1.36)	3.80 (1.04)	3.55 (1.20)	1–5	–
Self-esteem	28.77 (5.77)	29.14 (5.95)	27.78 (6.34)	28.76 (6.34)	10–40	0.89
Physical activity	4.18 (0.83)	4.17 (0.91)	4.15 (0.88)	4.17 (0.88)	1–5	

### Associations between SHCs and sex, age, time, and stress domains

Table III summarizes associations between sex, age, time point, and the outcome variable SHC. Sex was significantly associated with SHCs, with girls reporting higher SHC levels than boys. Age showed a significant curvilinear association with SHCs, with scores increasing from age 13 to 16, then declining from age 17 to 20. This was indicated by significant beta estimates for both age and age squared, as shown in Table III. Regarding time points, SHC scores were significantly higher in 2016 and 2022 compared to 2011.

**Table III.** Multiple linear regression analysis of associations between sex, age, time, and subjective health complaints.

Subjective health complaints			
	*B*	β	95% CI
Sex – girls ref.cat	–3.52	–.30***	–3.98/–3.06
Age	5.32	.10***	2.96/7.68
Age squared	–.16	.09***	–.24/–.09
Time – 2011 ref.cat			
2016	1.08	.09***	.58/1.59
2022	2.15	.13***	1.43/2.87

Note. * *p* < .05; ** *p* ⩽ .01; *** *p* ⩽ .001. *B*: unstandardized beta coefficient; β: standardized beta coefficient; CI: confidence interval. Age squared test Curvilinear association of age. *n* = 2,108 with listwise exclusion of cases. *R*^2^ = .13.

Table IV presents the results of two multiple linear regression models investigating associations between stress domains and SHCs. In Model 1, each stressor domain was tested individually while controlling for sex and age. All stressors were significantly and positively associated with SHCs, showing moderate strong associations. The explained variance (*R*^2^) for individual stressors ranged from .05% to 24%.

**Table IV.** Multiple linear regression analysis of associations between stressor domains and subjective health complaints.

Subjective health complaints						
	Model 1			Model 2		
	*B*	β	95% CI	*B*	β	95% CI
**Stress domains**						
Teacher/adult interaction (TI)	.30	.22***	.24/.36	.26	.17***	.41/.11
Peer pressure (PP)	.41	.34***	.36/.46	.05	.04	–.10/.20
Home life (HL)	.42	.32***	.37/.47	.07	.06	–.06/.21
Romantic relations (RR)	.19	.15***	.14/.24	–.02	–.02	–.14/.10
School attendance (SA)	.60	.37***	.54/.67	.40	.24***	.21/.58
School/leisure conflict (SLC)	.36	.27***	.30/.41	–.02	–.02	–.18/.14
School performance (SP)	.48	.34***	.42/.53	.12	.08*	.03/21
TI × sex				.11	.05	–.05/.27
PP × sex				–.04	.06	–.03/.26
HL × sex				–.08	–.01	–.16/.11
RR × sex				–.10	–.06	–.23/.01
SA × sex				–.26	–.16***	–.59/–.22
SLC × sex				–.06	.01	–.13/.16
SP × sex				–.08	.02	–.11/.21

Note. * *p* < .05; ** *p* ⩽ .01; *** *p* ⩽ .001. *B*: unstandardized beta coefficient; β: standardized beta coefficient; CI: confidence interval.

Model 1 showing estimates for each individual stressor, adjusted for sex and age. The sample size varies between *n* = 1,980 and *n* = 2,064, with listwise exclusion of cases. *R*^2^ varies between .05 and .24.

Model 2 shows estimates for all stressors, adjusted for sex, age, time, self-rated health, physical activity, and self-esteem. *n* = 1,810 with listwise exclusion of cases. *R*^2^ =.41.

In the fully adjusted Model 2, which included all stress domains and controlled for sex, age, time, self-rated health, physical activity, and self-esteem, three stressors remained significantly positively associated with higher SHCs: stress related to school attendance (β .24), teacher/adult interaction (β .17), and school performance (β .08). Interaction analysis revealed a significant interaction between sex and school attendance (Table IV), indicating a stronger association for girls. This means that as stress related to school attendance increases, the rise in SHCs is steeper for girls than for boys. No other interactions between sex by stress domains were statistically significant. Overall, Model 2 explained 41% of the variance in SHCs.

## Discussion

This study provides new insight into adolescents’ report of SHCs through three cross-sectional time-point assessments over 10 years, examining associations between sex, age, time, and normative stress domains. Three key findings emerged: (1) significant associations between sex, age, and SHCs; (2) relatively stable mean levels of SHCs over the three time points, with a slight increase observed over time; and (3) significant positive associations between stress domains, particularly those related to the school context, and SHCs, with a notable interaction effect between sex and stress related to school attendance.

Regarding the demographic variables, girls scored significantly higher on SHCs than boys. These results are in line with numerous previous findings [8, 11, 15, 18]. Girls’ consistently higher levels of SHCs could be attributed to differences in internalising behaviours, heightened interpersonal sensitivity, and greater self-reported emotional awareness [24, 25, 33], which are known to emerge during this period. These factors may make girls more prone to reporting psychosomatic symptoms in response to normative stressors. Furthermore, the curvilinear relationship between age and SHCs, where levels increased from ages 13 to 16 and then declined from 17 to 20, supports earlier research indicating that SHCs are common in adolescence and tend to rise during early to mid-adolescence [10–12]. This trajectory may reflect the increased exposure to developmental transitions, academic and social pressures, and identity formation during early adolescence, which can heighten stress and contribute to health complaints [18–20]. SHCs may decrease as adolescents enter late adolescence because roles become more defined and stable, autonomy increases, and coping mechanisms improve. In line with developmental stress theories, these findings may also reflect age-related improvements in emotion regulation and coping, and self-concept clarity, which typically strengthen through late adolescence [34].

The descriptive results showed that mean scores on SHCs were consistently slightly above the mid-point of the scale at all time points, with the highest scores observed in 2022, followed by 2016 and 2011. Consistently, the results of the regression analysis revealed a significant effect of time point on SHCs, with adolescents reporting higher SHCs in 2022 and 2016 compared to 2011. These findings are in line with previous national [11] and international [13, 15] studies, which show overall stability in adolescent SHCs, although there are slight increases in SHCs over time. The elevated levels of SHCs reported in 2022 may be partly attributed to the lingering effects of the COVID-19 pandemic [35]. Widespread social lockdowns had a profound impact on adolescents’ daily routines, limiting their interactions with peers and teachers and reducing access to important sources of social support and structure [35]. Such disruptions have been linked to increased emotional distress, particularly among adolescents who are developmentally sensitive to changes in peer relationships and daily routines [35, 36]. Notably, the somewhat increasing trend in adolescent SHCs is not only a national trend in Norway [11], but also a global phenomenon [4, 7, 13, 15]. However, the recent stabilization observed in Norway over the past five years, especially after the acute phases of the COVID-19 pandemic [2], may indicate that increased awareness of adolescent mental health or school-based support systems and getting back to daily life routines have helped to alleviate the negative impacts of the pandemic.

Regarding stressor domains, all stressors showed significant individual associations with SHCs after controlling for sex and age. In the model including all stressors, controlling for additional covariates, stress related to school attendance, teacher/adult interactions, and school performance retained significant positive associations with SHCs, with a stronger association found between school attendance and SHCs among girls. The adjusted model explained totally 41% of the variance in SHCs.

The impact of interpersonal stressors, such as peer pressure and romantic relationships, is especially relevant during adolescence because social identity becomes the core aspect of self-concept [18, 23]. As adolescents increasingly seek independence from their families and turn to peers for social and emotional connections and validation, conflicts within these relationships can significantly heighten their stress levels [21, 23].

The results of the fully adjusted regression model revealed that school-related stressors were significantly associated with SHC. These findings are consistent with the literature, which notes that school-related pressures are important sources of adolescent stress [9, 19, 23]. As adolescents progress through the educational system, they face increasing expectations related to academic achievement, adherence to rules and responsibilities, and interactions with teachers and peers [18, 23, 37]. While exposure to academic demands can support learning and development, it may also represent a significant source of stress during this life stage [9, 19, 23, 34]. Moreover, the school environment functions not only as an academic setting but also a social arena, where adolescents are continually evaluated and compared to their peers, which may heighten feelings of worry [23]. The interplay between academic stress and the social dynamics of school life may contribute to cumulative stress exposure and stress appraisal, especially when adolescents perceive limited control over, or insufficient support in coping with, these demands. Consequently, this may create a chronic sense of tension that can negatively impact health [18, 19]. The association between school-related stressors and SHCs, while controlling for relevant covariates, is consistent with previous research demonstrating that stress and SHCs are both prevalent and closely linked during adolescence [9, 18].

The strongest association between stress and SHCs was found in relation to school attendance, particularly among girls, as indicated by the significant interaction effect. Although girls often outperform boys academically, they also tend to report higher levels of school-related stress, which may may help explain the stronger association observed in girls [19, 23, 37]. This association may also reflect gender differences in how stress is appraised and managed. Research suggests that girls are more likely to internalize stress and engage in ruminative coping (repetitive thinking about distressing situations), which makes them more vulnerable to psychosomatic outcomes [24, 25, 33, 34]. However, most interaction effects between stress domains and sex were not statistically significant, suggesting that the overall relationship between stress and SHCs is largely consistent across sexes.

### Implications and practical applications

Given the associations found between sex, age, stressors (especially school-related stressors), and SHCs, a comprehensive and nuanced approach is essential when considering the public health implications. The results support the importance of strategies that are aware of stress exposure during adolescence and facilitate positive coping in adolescents’ daily life contexts (home/family, community, leisure time, and school). Adolescents spend much of their formative years in school, making it an important context for learning and health-promoting efforts [38]. Stress related to school attendance, academic performance, and teacher/adult interactions were all significantly associated with SHCs. These findings underscore the need for school-based initiatives that promote a positive learning environment and resilience in students, and support their development of socio-emotional skills. Encouraging adaptive coping strategies can also be an effective buffer for stress-induced SHCs [38]. Implementing such strategies relies on cross-sectorial collaboration and integration into different developmental contexts in adolescents’ lives.

### Strengths and limitations

This study benefits from a large sample size with cross-sectional data from three time points, allowing for examination of trends in adolescents’ reporting of SHCs and experience of stress over a 10-year period. The use of consistent measures across time points ensures reliable comparisons. However, the smaller sample size in 2022, due to COVID-19 restrictions, is a limitation. Additionally, slight variations in school participation across the assessment points may introduce sampling bias.

The data collections were conducted anonymously during a school hour, which helps reduce social desirability bias. In 2022, data were collected using a digital survey, while the data collection in 2011 and 2016 was conducted with paper-based questionnaires. Although digital surveys are cost-effective and easily distributed, they can be susceptible to technological issues that may impact data quality. Paper-based surveys offer more control over physical administration and are less prone to technological barriers but require more resources for implementation.

A key limitation is the substantial missing data on some variables. To address this, we used complete case analysis instead of methods like multiple imputation, which assumes that data are missing at random. Given that missingness was likely linked to specific variables, multiple imputation would have been unreliable in this case.

Another limitation is the use of self-reported data on subjective health, which can be influenced by social desirability or recall bias. However, the large sample size and consistent assessment across time help mitigate these potential biases. Finally, the cross-sectional design precludes conclusions about causal relationships. A longitudinal design could offer more insight into individual trajectories and within-person changes in SHCs over time.

## Conclusion

This study reports stable levels of SHCs in adolescents, with a minor increase in average levels over the 10 years. Girls reported higher levels of SHCs than boys, and a curvilinear association was found between age and SHCs. Stressors related to interpersonal interactions with adults and teachers, school attendance, and academic performance were positively associated with SHCs. These findings highlight the need for a comprehensive approach to adolescent stress, emphasizing school-based initiatives that focus on reducing academic and social stressors, fostering resilience, and promoting healthy coping mechanisms to help prevent or alleviate SHCs.
